# Combating Metallo-β-Lactamase-Producing *Pseudomonas aeruginosa*: The Fractional Inhibitory Concentration Index as a Tool to Evaluate Antibiotic Synergy

**DOI:** 10.3390/antibiotics14020210

**Published:** 2025-02-19

**Authors:** Guido Granata, Carolina Venditti, Claudia Rotondo, Valentina Dimartino, Silvia D’Arezzo, Assunta Gallo, Gabriella Parisi, Alessandro Capone, Carla Fontana, Stefania Cicalini

**Affiliations:** 1 Systemic and Immune Depression-Associated Infection Unit, National Institute for Infectious Diseases “L. Spallanzani”, IRCCS, Via Portuense 292, 00149 Rome, Italy; 2 Laboratory of Microbiology, National Institute for Infectious Diseases “L. Spallanzani”, IRCCS, 00149 Rome, Italy; 3 Laboratory of Microbiology, S. Camillo Hospital, 00149 Rome, Italy

**Keywords:** carbapenem-resistant *Pseudomonas aeruginosa*, metallo-β-Lactamase, IMP-13 carbapenemases, antimicrobial synergy, cefiderocol, imipenem–relebactam, bloodstream infection, antimicrobial stewardship, antibiotic treatment, Verona integron–encoded metallo-β-lactamase, VIM

## Abstract

**Background**: Multi-drug-resistant Gram-negative bacteria producing metallo-β-lactamase are an increasing concern. Here, we described three cases of infection due to difficult-to-treat and drug-resistant *P. aeruginosa* producing metallo-β-lactamases, which were successfully treated with antibiotic combination of cefiderocol plus imipenem–relebactam, and reported on the molecular and epidemiological features of the isolates and the in vitro synergistic effects of different antibiotic combinations guiding antibiotic treatment. **Patients and methods**: Three *P. aeruginosa* strains were isolated from respiratory or blood cultures of three different patients. Minimum inhibitory concentrations breakpoints were interpreted according to EUCAST recommendations. Next-generation sequencing data were used for in silico identification of resistance genes and sequence types and for core genome multi-locus sequence typing analysis. The fractional inhibitory concentration index was performed as a measure of synergy of cefiderocol plus imipenem and imipenem–relebactam. **Results**: The three isolates exhibited different multi-drug-resistant and molecular profiles carrying blaIMP-13 (imipenemase metallo-β-lactamase-13) (isolates named Pse-1 and Pse-3) and blaVIM-2 (Verona integron-encoded metallo-β-lactamase-2) (isolate Pse-2). Typing showed that the isolates did not cluster and belonged to different sequence types. The E-test method showed the presence of synergy of cefiderocol in combination with imipenem–relebactam in the two *P. aeruginosa* isolates producing IMP-13 (Pse-1 and Pse-3). No synergy was observed in the isolate producing VIM-2 (Pse-2). **Conclusions**: Cefiderocol in association with imipenem–relebactam exhibited a synergistic effect against IMP-producing *P. aeruginosa* isolates. Further studies with a range of drugs and an expanded number of isolates are required to ascertain potential novel synergistic associations and the clinical utility of the fractional inhibitory concentration index.

## 1. Introduction

The Gram-negative bacterium *Pseudomonas aeruginosa* is a major cause of hospital-acquired infections. Frail hospitalized patients such as those who have suffered burns, require ventilation, or have neutropenia or chronic debility, are at higher risk of developing infections due to *P. aeruginosa*. *P. aeruginosa* possesses intrinsic resistance and may acquire mechanisms conferring resistance to a variety of antibiotics, including extended-spectrum lactamases (ESBLs) and carbapenemases.

Worldwide, difficult-to-treat (DTR) *P. aeruginosa* causes a wide range of severe infections, including pneumonia, bloodstream infections (BSI), endophthalmitis, endocarditis, and meningitis [1,2]. DTR *P. aeruginosa* is frequently associated with high mortality and morbidity rates [1,2].

A common feature of DTR and pan-drug-resistant *P. aeruginosa* isolates is the production of metallo-β-lactamases (MBLs) such as Verona integron–encoded (VIM) and active on imipenem (IMP) metallo-β-lactamase. A great variety of ESBLs is also reported, such as oxacillinase (OXA), Pseudomonas extended resistance β-lactamase (PER), Guiana extended-spectrum β-lactamase (GES), Vietnam extended-spectrum β-lactamase (VEB), and polyamine oxidase (PAO) β-lactamase [3].

Nowadays, β-lactamase enzymes are widely disseminated among different *P. aeruginosa* sequence types (STs) and are often associated to other resistance mechanisms, including decreased membrane permeability or active efflux pump systems [4,5,6]. Since the year 2000, a persistent circulation of DTR *P. aeruginosa* strains producing VIM and IMP has been reported in Italian hospitals, frequently resulting in outbreaks with limited treatment options [7,8,9]. As novel antimicrobial compounds such as ceftolozane–tazobactam, ceftazidime–avibactam, and imipenem–relebactam are useful against non-MBL producer *P. aeruginosa*, cefiderocol might be considered a last resort antibiotic for MBL-producing strains [2,9].

Here, we reported on the management of three cases of severe infections caused by DTR, MBL-producing *P. aeruginosa* strains and investigated the molecular and epidemiological features of the isolates as well as the in vitro synergistic effects of different antibiotic combinations.

## 2. Results

### 2.1. Clinical Cases Presentation

Three patients were included in the study, receiving combination antibiotic treatment with cefiderocol and imipenem/relebactam. All three patients had shown prompt clinical improvement and microbial eradication without cases of relapse, resulting in their discharge from the intensive care units.

#### 2.1.1. Case 1—Isolate Pse-1

The first case was that of a 65-year-old patient who had undergone cardiac surgery to replace the ascending aorta due to a diagnosis of type A dissection. The patient developed post-operative cardiac tamponade, causing hypotension and acute renal failure. Surgical revision was required, after which the patient was transferred to the intensive care unit for continuous renal replacement therapy. During their subsequent stay in the intensive care unit, the patient was diagnosed with mechanical ventilator-associated pneumonia. The microorganism isolated from bronchoalveolar lavage was a *P. aeruginosa* producing IMP-13 MBL (Pse-1). The patient was treated with a combination antibiotic treatment with cefiderocol (2 g every 8 h, as a continuous infusion) in association with imipenem/relebactam (0.625 g every 6 h, for a total duration of 7 days). This resulted in a rapid resolution of the clinical picture, allowing for a rapid respiratory weaning with early extubation four days after the start of combination antimicrobial therapy.

#### 2.1.2. Case 2—Isolate Pse-2

The second case concerned a central venous catheter-related bloodstream infection in a 37-year-old patient who had been hospitalized in the intensive care unit due to a recent road polytrauma. A strain of *P. aeruginosa* producing VIM was isolated from peripheral blood cultures and from the central venous catheter. The blood from the central venous catheter demonstrated microbial growth 3 h earlier than the blood simultaneously collected from a peripheral vein (Pse-2). The patient underwent replacement of the central venous catheter, and a combination antibiotic treatment was started, comprising cefiderocol (2 g every 8 h in extended infusion) and imipenem/relebactam (1.250 g every 6 h for a total duration of 14 days). During this period, the patient recovered rapidly. Control blood cultures were negative, and the echocardiogram revealed no evidence of cardiac vegetation.

#### 2.1.3. Case 3—Isolate Pse-3

The third case was a 43-year-old woman with bacteremic pneumonia associated with mechanical ventilation. The patient was initially admitted to the intensive care unit for the treatment of pneumonia and severe acute respiratory failure caused by the influenza A H1N1 virus. A strain of *P. aeruginosa* producing IMP-13 was isolated from the blood (Pse-3). The patient was treated with a combination of cefiderocol (2 g every 8 h in prolonged infusion) and imipenem/relebactam (1.250 g every 6 h for a total duration of 14 days). The patient recovered rapidly. Control blood cultures were negative and the respiratory exchanges progressively improved, allowing extubation three days after starting the combination therapy.

### 2.2. Phenotypic and Molecular Characterization of Isolates

As shown in Table 1, the three isolates (named Pse-1, Pse-2, and Pse-3) were obtained from different specimens: bronchoalveolar lavage for Pse-1 and blood culture for Pse-2 and Pse-3. Pse-1 and Pse-2 were resistant to carbapenems, ceftazidime–avibactam, ceftolozane–tazobactam, meropenem–vaborbactam, imipenem–relebactam, and ciprofloxacin. Isolate Pse-3 was resistant to ceftazidime–avibactam and ceftolozane–tazobactam. All three isolates were susceptible to cefiderocol.

Detection of acquired antimicrobial resistance genes and STs are recorded in Table 2.

The three isolates were positive for beta-lactams resistance genes such as MBLs (VIM-2 or IMP-13), blaPAO, and OXA-type beta-lactamases, but belonged to different STs.

Pse-1 (ST621) harbored blaIMP-13. Pse-2 instead produced a VIM-2 carbapenemase and belonged to the ST233, a clonal strain, referred as an international high-risk clone, responsible for nosocomial infections identified worldwide [10]. Additional resistance profiling of Pse-2 isolates showed the presence of three aminoglycoside resistance genes [aac(6′)-II, aph(3′)-IIb, and aac(3′)-Id], three phenicol resistance genes [catB7, cmlA1, and floR], a quaternary ammonium compound qacE, and tetracycline, fosfomycin, sulfonamide, trimethoprim, and quinolone resistance genes tet(G), fosA, sul1, dfrB5, and crpP, respectively.

**Figure 1:**
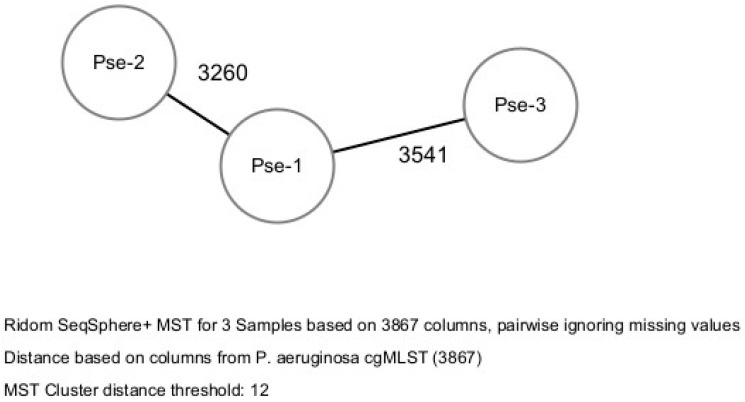
Bacterial epidemiological typing of the three isolates.

Bacterial epidemiological typing was performed by the whole genome sequencing cgMLST. A gene-by-gene approach with 3867 target genes was used to compare the genomes. The allelic distance between the three isolates was greater than the threshold of 12 allelic differences, therefore no clusters were identified. Therefore, according to the cgMLST method, the three isolates did not cluster into a cluster-type and were categorized as belonging to three STs showing high allelic distance (more than 3000 allele difference) (Figure 1).

### 2.3. The Evaluation of Antibiotic Synergy by Fractional Inhibitory Concentration Index (FICI)

The evaluation of antibiotic synergy by the E-test method and the FICI showed the presence of synergy/additivity of cefiderocol in combination with imipenem–relebactam in the two *P. aeruginosa* isolates producing IMP-13. Pse-1 had a FICI of 0.34 (synergy), Pse-3 had a FICI of 0.84 (additivity). No synergy between cefiderocol and imipenem–relebactam was observed for the Pse-2 isolate producing VIM-2 (FICI of 2, defined as indifference) (Table 3).

Bacterial epidemiological typing was performed by the whole genome sequencing-based core genome multi-locus sequence typing (cgMLST). A gene-by-gene approach with 3867 target genes was used to compare the genomes.

## 3. Discussion

The global occurrence of carbapenem-resistant and MDR *P. aeruginosa* is alarming because infections by these bacteria often result in limited treatment options [11]. The antimicrobial cefiderocol might be considered a last resort drug for some MBLs such as non-New Delhi metallo-β-lactamase-producing *P. aeruginosa* strains. Alarmingly, the emergence of cefiderocol resistance has been reported in non-New Delhi metallo-β-lactamase producing *P. aeruginosa* strains [12,13]. The emergence of resistance to cefiderocol in *P. aeruginosa* has been demonstrated both in vitro and in vivo, and linked to alterations of the iron uptake pathways [14,15,16,17].

Here, we present three cases of severe infection sustained by MBL-producing *P. aeruginosa* strains, successfully treated with an antibiotic combination of cefiderocol and imipenem/relebactam. We acknowledge that the small sample size represents a major study limitation, and we are planning to include more cases in future studies, despite the challenges in enrolling severe cases of infections due to MBL-producing *P. aeruginosa*. Another limitation of the study is the lack of long-term data after the antibiotic treatment.

Also, it is important to remark that currently there is no clear evidence that combination therapy is more effective than using a single antibiotic to treat infections due to MDR *P. aeruginosa*, unless perhaps in severe cases like septic shock [18].

A recent multicenter, retrospective cohort study [19] compared the outcomes of patients with septic shock due to *P. aeruginosa* BSI receiving either adequate empirical combination therapy or adequate empirical monotherapy. Adequate empirical combination therapy was associated with a lower 30-day all-cause mortality (25%, six out of 24) compared to adequate empirical monotherapy (56.8%, 42 out of 74; *p* = 0.007). Multivariate Cox regression analysis indicated adequate empirical combination therapy as the only factor significantly associated with improved survival (aHR 0.30; 95% CI 0.12–0.71; P: 0.006) [19].

The administration of cefiderocol in combination with other antibiotics, i.e., ceftazidime/avibactam, ampicillin/sulbactam, or meropenem, has been recently proposed to treat MDR and pan-drug-resistant Gram-negative bacteria, including *P. aeruginosa* [20,21]. The rationale for administering cefiderocol in combination with other antimicrobials is to avoid resistance development and to exploit a potential synergistic effect [20].

The antibiotic combination may provide a synergistic effect, determining a greater and faster bactericidal effect, with improved patient outcome [22]. In addition, a potential benefit of the combination therapy is the reduction in the emergence of resistance to cefiderocol [23,24]. To consider, combination therapy may also have disadvantages, such as potentially increasing toxicity or increased C. difficile infections [25]. Moreover, there is currently little data available to guide the choice of ancillary drug to be given with cefiderocol in the treatment of MBL-producing *P. aeruginosa* infections, i.e., fosfomycin or carbapenems.

In the literature, there is scant data on the evaluation of synergy between cefiderocol and other antimicrobials against *P. aeruginosa* [26,27].

Therefore, an assay to evaluate the synergy between cefiderocol and imipenem, imipenem/relebactam, fosfomycin, as well as other antibiotics, may have potential therapeutic utility in the management of MBL-producing *P. aeruginosa* infections to assist in the selection or discontinuation of the companion drug in combination with cefiderocol. Of course, this approach may only be useful if supported by prior microbiological testing of the isolate susceptibility profile and molecular characterization. To our knowledge, this is the first report using the E-test technique and FICI to evaluate the synergy of cefiderocol plus imipenem/relebactam against MBL-producing *P. aeruginosa* strains.

By E-test and FICI evaluation, our two *P. aeruginosa* producing IMP-13 MBL showed synergy or additivity of cefidercol plus imipenem/relebactam, whilst the isolate producing VIM-2 showed no synergy effect. The mechanisms behind the synergy between cefiderocol and imipenem against *P. aeruginosa* are still unclear. One possible explanation could be the inhibition of multiple PBP targets by the two drugs (i.e., PBP 3, which is preferentially bound by cefiderocol, and other PBPs including PBP2, which are bound by imipenem at concentrations that might partially evade degradation by MBLs) [27,28,29].

Further studies on the potential synergistic effects of cefiderocol combinations with carbapenems and other antimicrobials would be useful, and the FICI evaluation may be a useful tool to evaluate antibiotic synergy. Moreover, in the future, the FICI values and the E-test technique may be useful tools to support clinicians in the choice of the most effective antibiotic treatment, even for other that MBL-producing *P. aeruginosa* strains. 

Understanding of the specific MBL type may also be of potential benefit. In our study, the difference in the phenotypic profiles of the three *P. aeruginosa* isolates could be a result of the difference in the resistome. All isolates were positive for β-lactam resistance genes such as MBLs (VIM or IMP), blaPAO, and an OXA-type beta-lactamase, and belonged to three different STs. Pse-1 and Pse-3, which were ST621 and ST446, respectively, harbored blaIMP-13 and an identical additional resistance profile. Interestingly, they showed a completely different phenotypic profile. This could be due to a difference in membrane permeability and efflux pumps, as both OXA-50 and OXA-395 are oxacillinases with weak carbapenemase activity (unique difference in the molecular profile of isolates Pse-1 and Pse-3) [6,30].

## 4. Patients and Methods

In January–February 2024, three cases of infection due to MBL-producing *P. aeruginosa* were diagnosed in three different intensive care units at the San Camillo Hospital in Rome. Three *P. aeruginosa* strains were isolated from respiratory or blood cultures from the three patients. The samples were collected before the start of a combination antibiotic treatment and were sent to the Microbiology Laboratory of the National Institute for Infectious Diseases “L. Spallanzani”, IRCCS Microbiology Laboratory for further phenotypic and molecular characterization of the isolates and to evaluate in vitro antibiotic synergy. The three patients included in the study received a combination antibiotic treatment consisting of cefiderocol and imipenem/relebactam. The treatment was effective in all three patients, with prompt clinical improvement and microbial eradication. No relapses of *P. aeruginosa* infection were observed during a six-week follow-up starting from the end of the combination antibiotic treatment.

### 4.1. Phenotypic and Molecular Characterization of Isolates

Antibiotic susceptibility and species identification were determined by the Vitek-2 System (bioMérieux, Marcy l’Étoile, France), AST-438 plus XZ26, and MALDI-TOF MS Biotyper Sirius (Bruker Daltonics, Fahrenheitstraße, Bremen, Germany), respectively. Minimum inhibitory concentrations (MICs) for cefiderocol were performed via broth microdilution and synergy testing by gradient strip method (Liofilchem, Roseto degli Abruzzi, Italy). The medium used for the microdilution broth test assay was Mueller Hinton cation-corrected and iron-depleted broth (ID-CAMHB) supplied directly from the Liofilchem’s kit. Results were interpreted according to the European Committee on Antimicrobial Susceptibility Testing [31]. First, detection and identification of the most diffused carbapenemases (KPC, OXA-48-like, IMP, VIM and NDM) were achieved using immunochromatographic assay (NG-Test CARBA 5, Biotech, France) and confirmed by whole genome sequencing (WGS) performed by Illumina Miseq (San Diego, CA, USA). All raw reads generated were submitted to the Sequence Read Archive (SRA) under the BioProject ID PRJNA1161848.

The resistance profile and sequence types (STs) of isolates were identified by in silico analysis using the ResFinder v3.0 web server [32]. Bacterial epidemiological typing was performed by the WGS-based core genome MLST (cgMLST) scheme v1.0, using the Ridom SeqSphere+ software, version 9 (Ridom GmbH, Münster, Germany) with default settings. Based on the defined cgMLST for *P. aeruginosa*, a gene-by-gene approach with 3867 target genes was used to compare the genomes [33]. According to the manufacturer’s instructions, compared to the reference strain (GenBank accession no. NC_002516.2), the resulting set of target genes was then used for interpreting the clonal relationship displayed in a minimum spanning tree (MST) using a complex type (CT) distance of 12 alleles [34].

### 4.2. The Fractional Inhibitory Concentration (FIC) Index as a Measure of Antibiotic Synergy

An inoculum equal to 0.5 McFarland turbidity was prepared from each isolate. The turbidity value of the standard of 0.5 MFU (McFarland Units) corresponds to approximately a culture density of 1.5 × 10^8^ cells/mL. The turbidity was measured using VITEK^®^ DENSICHEK (Biomerieux, Commune, France). Determination of MICs by E-test was first performed for the two drugs separately, and the MICs were interpreted at the point of intersection between the inhibition zone and the E-test strip. For synergy testing, the two E-test strips were placed on the same culture plate in a cross formation so that they intersected each other at their respective MICs at a 90° angle or at the highest concentration present on the E-test strip when the MIC exceeded this value (e.g., >256 mg/L). The plates were then incubated at 37 °C for 24 hours. The Fractional Inhibitory Concentration Index (FICI) was calculated on the basis of the resulting zone of inhibition as follows: FICI=FIC A+FIC B, where FIC A is the MIC of the combination/MIC of drug A alone and FIC B is the MIC of the combination/MIC of drug B alone [35]. Interpretation of the FIC results, according to accepted criteria, were as follows: ≤0.5: synergy; 0.5 to 1.0: additivity; >1.0 to 4.0: indifference; and >4: antagonism [35].

## 5. Conclusions

Until further data are available, clinicians may consider combination therapy with cefiderocol and another antimicrobial for the treatment of severe infections caused by MBL-producing *P. aeruginosa* in order to avoid the development of cefiderocol resistance and to take advantage of a potential synergistic effect.

The E-test and FICI evaluation may be valuable tools in the treatment of MBL-producing *P. aeruginosa* infections, assisting in the selection or discontinuation of the companion antibiotic in combination with cefiderocol.

## Figures and Tables

**Table 1:** Antimicrobial susceptibility results for clinical isolates described in this study.

Strain	Sample	Date	MBL	Antibiotic MIC Values (mg/L)										
				CZA	FDC	C/T	M/V	I/REL	IPM	MEM	CAZ	CIP	FOS	PZT
Pse-1	BAS	02.01.24	IMP-13	>256, R	0.064, S	>256, R	32, R	>32, R	>32, R	≥16, R	≥64, R	≥4, R	16, S	8, SI
Pse-2	Blood	26.01.24	VIM-2	>256, R	0.19, S	>256, R	≥64, R	>32, R	>32, R	≥16, R	≥64, R	≥4, R	>256, SI	≥128, R
Pse-3	Blood	26.01.24	IMP-13	>256, R	0.047, S	>256, R	2, S	1.5, S	2, SI	4, SI	≥64, R	0.25, SI	6, S	8, SI

CZA, ceftazidime/avibactam; FDC, cefiderocol; C/T, ceftolozane/tazobactam; M/V, meropenem/vaborbactam; I/REL, imipenem/relebactam; IPM, imipenem; MEM, meropenem; CAZ, ceftazidime; CIP, ciprofloxacin; FOS, fosfomycin; PZT, piperacillin/tazobactam; S, susceptible; R, resistant; SI, susceptible high dosage.

**Table 2:** Molecular characterization of clinical isolates.

Strain	Genetic Determinants		Typing
	Beta-Lactam	Additional Resistance Genes	ST
Pse-1	*bla*_IMP-13_, *bla*_OXA-50,_*bla*_PAO_	*aph(3′)-IIb*, *qacE*, *fosA*, *catB7*, *crpP*, *sul1*	621
Pse-2	*bla*_VIM-2_, *bla*_OXA-486,_*bla*_PAO_	*aac(6′)-II*, *aph(3′)-IIb*, *aac(3′)-Id*, *qacE*, *fosA*, *catB7*, *cmlA1*, *floR*, *crpP*, *sul1*, *tet(G)*, *dfrB5*	233
Pse-3	*bla*_IMP-13_, *bla*_OXA-395,_*bla*_PAO_	*aph(3′)-IIb*, *qacE*, *fosA*, *catB7*, *crpP*, *sul1*	446

ST: Sequence type identified by multi-locus sequence typing method.

**Table 3:** The measure of antibiotic synergy by fractional inhibitory concentration index (FICI). Interpretation of the FIC results were as follows: ≤0.5, synergy; 0.5 to 1.0, additivity; >1.0 to 4.0, indifference; and >4, antagonism.

**Pse-1 (IMP-13 Producer)**	**Agar Diffusion (E-Test) MIC**	**Fractional Inhibitory Concentration Index**
Cefiderocol + Imipenem	Cefiderocol alone: 0.064 Cefiderocol combination: 0.016 Imipenem alone: >32 Imipenem combination: 2	0.31
Cefiderocol + Imipenem/relebactam	Cefiderocol alone: 0.064 Cefiderocol combination: 0.016 Imipenem/relebactam alone: >32 Imipenem/relebactam combination: 3	0.34
Cefiderocol + Fosfomycin	Cefiderocol alone: 0.064 Cefiderocol combination: 0.016 Fosfomycin alone: 16 Fosfomycin combination: 6	0.62
**Pse-2 (VIM-2 Producer)**	**Agar Diffusion (E-Test) MIC**	**Fractional Inhibitory Concentration Index**
Cefiderocol + Imipenem	Cefiderocol alone: 0.19 Cefiderocol combination: 0.19 Imipenem alone: >32 Imipenem combination: 32	2
Cefiderocol + Imipenem/relebactam	Cefiderocol alone: 0.19 Cefiderocol combination: 0.19 Imipenem/relebactam alone: >32 Imipenem/relebactam combination: 32	2
Cefiderocol + Fosfomycin	Cefiderocol alone: 0.19 Cefiderocol combination: 0.19 Fosfomycin alone: >256 Fosfomycin combination: 256	2
**Pse-3 (IPM-13 Producer)**	**Agar diffusion (E-Test) MIC**	**Fractional Inhibitory Concentration Index**
Cefiderocol + Imipenem	Cefiderocol alone: 0.047 Cefiderocol combination: 0.016 Imipenem alone: 2 Imipenem combination: 0.5	0.59
Cefiderocol + Imipenem/relebactam	Cefiderocol alone: 0.047 Cefiderocol combination: 0.016 Imipenem/relebactam alone: 1.5 Imipenem/relebactam combination: 0.75	0.84
Cefiderocol + Fosfomycin	Cefiderocol alone: 0.047 Cefiderocol combination: 0.023 Fosfomycin alone: 6 Fosfomycin combination: 2	0.81

## Data Availability

Data are contained within the article.
